# Autophagy-lysosome pathway dysfunction in MASLD progression: molecular mechanisms and therapeutic targets

**DOI:** 10.3389/fmed.2026.1961366

**Published:** 2026-10-14

**Authors:** Shuaibing Cao, Xinyue Cheng, Yi Yang, Baoping Lu, Shan Li, Chuntian Huang, Mingyu Ba, Lei Luo, Changpu Zhao

**Affiliations:** 1 The Second Clinical Medical College, Henan University of Chinese Medicine, Zhengzhou, China; 2 Medical College, Henan University of Chinese Medicine, Zhengzhou, China; 3 Henan Medical College, Zhengzhou, China; 4 The First Affiliated Hospital of Henan University of TCM, Zhengzhou, China; 5 The Second Affiliated Hospital of Henan University of TCM, Zhengzhou, China

**Keywords:** autophagy-lysosome pathway, lysosomal dysfunction, metabolic dysfunction-associated steatotic liver disease, metabolism, targeted therapy

## Abstract

Metabolic dysfunction-associated steatotic liver disease (MASLD) is the most prevalent chronic liver disease worldwide, yet no standardized pharmacotherapy has been approved for clinical use. The autophagy–lysosome pathway (ALP) serves as an essential intracellular degradation and recycling machinery that sustains hepatic metabolic homeostasis via modulation of lipid turnover, mitochondrial quality surveillance, and hepatocellular detoxification. Accumulating evidence demonstrates that ALP impairment acts as a core pathogenic factor across all stages of MASLD progression. This review systematically elaborates the molecular framework of the ALP and its multifaceted physiological functions in the liver, with special attention to the central pathogenic mechanisms underlying ALP impairment in MASLD: lysosomal dysfunction, defective autophagosome–lysosome fusion, dysregulation of key transcriptional and epigenetic regulators, and impaired subtypes of selective autophagy. ALP activity displays stage-specific characteristics across MASLD progression, featuring compensatory activation in simple hepatic steatosis but functional suppression in metabolic dysfunction-associated steatohepatitis (MASH). Hepatocytes and hepatic stellate cells exert opposing autophagic activity during liver fibrogenesis. We further summarize existing therapeutic strategies targeting the ALP, which fall into three categories: pharmacological agents (ALP agonists, lysosomal modulators, natural bioactive compounds, and nano-drug delivery systems), non-pharmacological interventions (dietary adjustment, physical exercise, and lifestyle modification), and gut–liver axis regulatory approaches. Finally, we address the primary translational bottlenecks and prospective avenues for converting these mechanistic findings into viable clinical therapies.

## 1 Introduction

Metabolic dysfunction-associated steatotic liver disease (MASLD) is the most prevalent chronic liver disease worldwide. It is strongly correlated with metabolic comorbidities including obesity, type 2 diabetes mellitus and hyperlipidaemia, it imposes a substantial global public health burden and affects approximately 38% of adults, with prevalence still rising (1, 2) MASLD progresses along a well-defined pathological spectrum from simple hepatic steatosisto metabolic dysfunction-associated steatohepatitis (MASH), fibrosis, cirrhosis and hepatocellular carcinoma (HCC) (3). To date, its pathogenic cascade remains incompletely characterized and no disease-specific pharmacotherapy hasbeen approved. Deciphering its core molecular mechanisms of MASLD and uncovering actionable therapeutic targets carries vital clinical and translational research significance.

The autophagy-lysosome pathway (ALP) represents a highly conserved intracellular catabolic cascade. It sustains cellular homeostasis by sequestering misfolded protein aggregates, impaired organelles and surplus lipids within autophagosomes, followed by autophagosome-lysosome fusion to facilitate substrate degradation (4). As the central organ governing whole-body metabolism, the liver depends on the ALP to orchestrate hepatic lipid turnover, mitochondrial quality surveillance, xenobiotic detoxification and tissue injury repair. Accumulating evidence links ALP impairment to the initiation and progression of MASLD. Notably, disrupted autophagic flux stemming from lysosomal dysfunction may act as a critical driver of the transition from simple hepatic steatosis to MASH (5). However, current literature predominantly centers on upstream regulation of autophagosome biogenesis while underappreciating the contribution of lysosomal defects and stage-dependent ALP dynamics across the full MASLD spectrum (6, 7).

Recent advances have shifted research from superficial phenotypic observations to detailed mechanistic dissection at the molecular scale, uncovering subunit-specific defects in V-ATPase (ATP6V1B2 and ATP6V0A1) as critical drivers of impaired lysosomal acidification (8); NSD2-mediated epigenetic repression of transcription factor EB (TFEB) as a novel regulatory signaling axis (9); LAMTOR1 has been validated as a direct molecular target of natural bioactive compounds such as acacetin (10). ALP activity also undergoes dynamic alterations that are both stage-dependent and cell-type-dependent dynamic alterations during disease progression: it is compensatory in hepatocytes in early simple steatosis but profoundly suppressed in MASH. Separately, autophagic activity is defective in hepatocytes yet elevated to drive profibrogenic signaling in hepatic stellate cells (11). Novel crosstalk between ALP impairment and ferroptosis has emerged as a synergistic pathogenic cascade linking dysregulated iron metabolism, lipid peroxidation, and sustained oxidative stress (12).

This review systematically synthesizes the physiological functions of the ALP in hepatic homeostasis and the pathogenic consequences of its impairment across the full spectrum of MASLD. We delineate the core molecular mechanisms driving ALP malfunction, including lysosomal impairment, defective autophagosome-lysosome fusion, dysregulated transcriptional/epigenetic regulators and aberrant selective autophagy, with emphasis on newly uncovered V-ATPase subunit-specific alterations and epigenetic regulatory circuits, and characterize stage- and cell-type-dependent dynamic shifts in ALP activity during disease progression. Finally, we critically assess existing ALP-targeted therapeutic modalities, encompassing pharmacological agents, natural bioactive compounds, nano-drug delivery systems, non-pharmacological lifestyle interventions, and gut-liver axis modulatory strategies. We also outline translational priorities for converting these mechanistic discoveries into viable clinical therapeutic regimens.

## 2 The complete process of the autophagy-lysosome pathway

The ALP constitutes a highly ordered, multistep synergistic intracellular degradation pathway. Its complete cascade is divided into four sequential, tightly regulated phases: autophagy initiation, phagophore nucleation and elongation, autophagosome-lysosome fusion, and lysosomal degradation (Figure 1). This pathway is not only responsible for clearing long-lived proteins, misfolded protein aggregates and damaged organelles, but also mediates the selective degradation of lipid droplets (i.e., lipophagy), and it occupies a central position in cellular energy homeostasis, protein quality control and stress adaptation (13).

**Figure 1:**
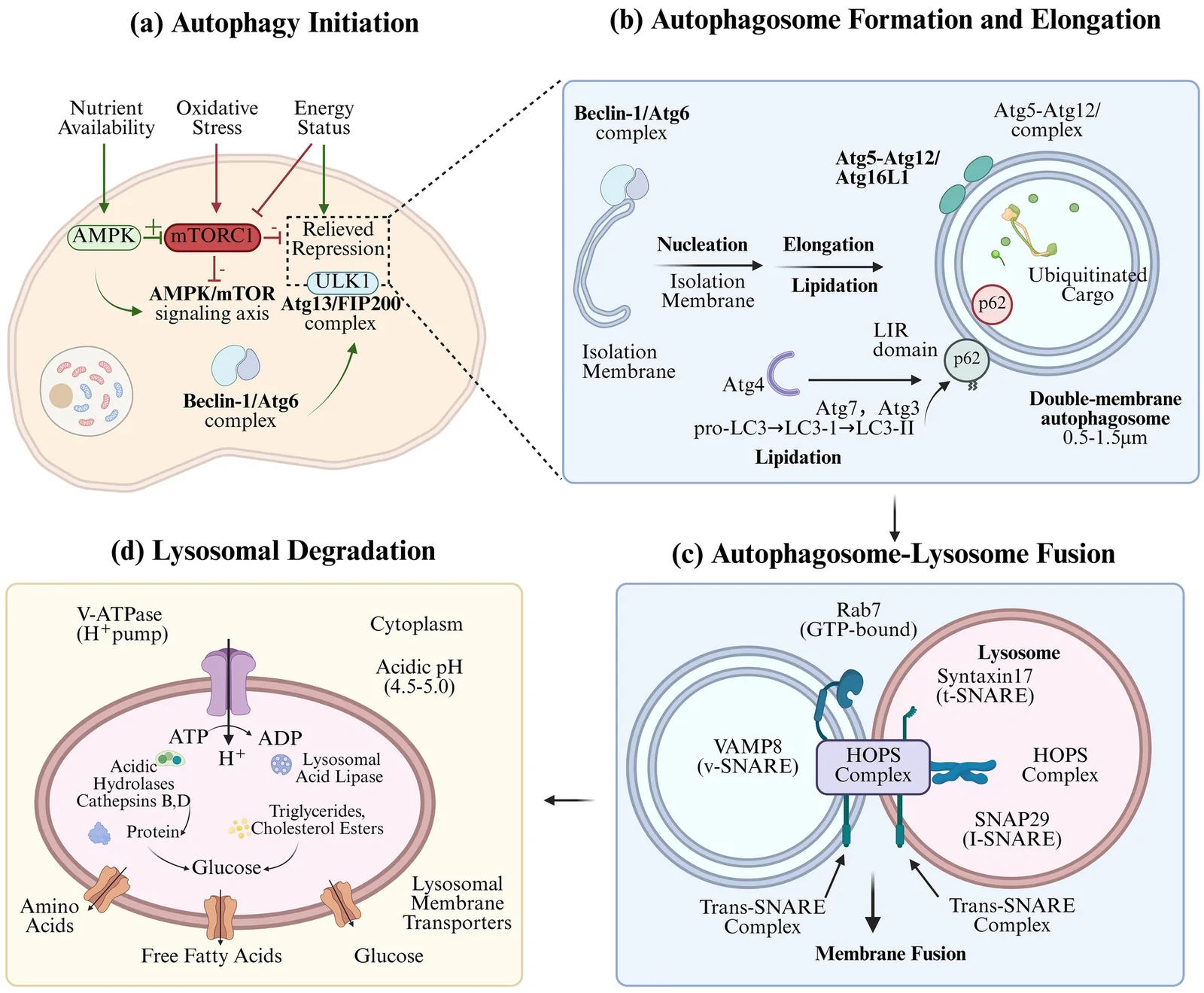
Schematic diagram of the complete ALP. **(a)** Autophagy initiation: in response to nutrient availability, oxidative stress and energy status, the AMPK/mTOR signaling axis regulates the ULK1/Atg13/FIP200 complex and the Beclin-1/Atg6 complex. **(b)** Autophagosome formation and elongation: nucleation of the isolation membrane and LC3 lipidation (pro-LC3→LC3-I→LC3-II) mediated by Atg4, Atg7 and Atg3, together with p62-mediated engulfment of ubiquitinated cargo, lead to the formation of the double-membrane autophagosome (0.5-1.5 μm). **(c)** Autophagosome-lysosome fusion: GTP-bound Rab7, the HOPS complex and SNARE proteins (YAMP8, Syntaxin17, SNAP29) mediate membrane fusion through the trans-SNARE complex. **(d)** Lysosomal degradation: the V-ATPase maintains an acidic pH (4.5-5.0); acidic hydrolases (cathepsins B, D) and lysosomal acid lipase degrade proteins, triglycerides and cholesterol esters, and the degradation products (amino acids, free fatty acids, glucose) are released into the cytoplasm via lysosomal membrane transporters. Created in BioRender. Cheng, X. (2026) https://BioRender.com/xlrmr3k.

The initiation of autophagy is mainly regulated by the AMP-activated protein kinase (AMPK)/mammalian target of rapamycin (mTOR) signaling pathway: Under nutrient deprivation or oxidative stress conditions, AMPK inhibits mTORC1 and relieves its inhibitory effect on the ULK1/Atg13/FIP200 complex, thereby triggering this first-order reaction (14–16). Subsequently, the formation and extension of autophagosomes involve the lipid modification of Atg family proteins and LC3: Beclin-1/Atg6 complex promotes phagosome nucleation, while Atg5-Atg12-Atg16L1 complex promotes autophagosome extension. Microtubule-associated protein 1 light chain 3 (LC3) is cleaved to form LC3-II, which anchors to the autophagosomal membrane. In this process, sequestosome 1 (p62/SQSTM1) recognizes ubiquitinated cargos (e.g., lipid droplets and misfolded proteins) and delivers these substrates into autophagosomes (17–20). Next, autophagosome-lysosomal fusion is regulated by SNARE proteins, Rab7 and HOPS complex: the v-SNARE protein VAMP8 on the autophagosome is paired with the t-SNARE protein synapticin 17 and SNAP29 on the lysosome to form a trans-snare complex, while the HOPS complex binds Rab7 to promote assembly and fusion (21, 22). If the fusion process is defective, it will hinder the autophagic flow and lead to the accumulation of goods. Finally, lysosomal degradation depends on the acidic compartment (pH 4.5–5.0) produced by vacuolar H^+^-ATPase (V-ATPase), as well as lysosomal hydrolases (such as lysosomal acidic lipase (LAL), cathepsin B and cathepsin D for lipid degradation). Degradation products (amino acids, free fatty acids (FFAs), glucose) are recycled to the cytoplasm through lysosomal membrane transporters to complete the material cycle (23–25).

## 3 Roles of the autophagy-lysosome pathway in hepatic physiological metabolism

As a central organ regulating systemic metabolism, the liver is responsible for coordinating the turnover of lipids, proteins, carbohydrates and other metabolic substrates. Under this physiological framework, ALP plays an indispensable role in maintaining liver homeostasis by regulating liver lipid turnover, maintaining mitochondrial integrity, and mediating detoxification of exogenous substances and tissue damage repair (7).

### 3.1 Regulation of hepatic lipid metabolism

The liver is the core organ of lipid synthesis and catabolism in the human body and plays an irreplaceable regulatory role in maintaining systemic lipid homeostasis. Under physiological conditions, hepatocytes synthesize lipids by ingesting fatty acids and *de novo* synthesis of lipids, and store the lipids in lipid droplets or export them as lipoproteins. However, excessive dietary lipid intake or systemic metabolic dysfunction can disrupt the balance between lipid production and clearance in hepatocytes, leading to abnormal lipid droplet deposition, which constitutes the basic pathological mechanism of MASLD.

In this context, ALP-mediated lipophagy has become a key mechanism for hepatocytes to remove excess lipid droplets. As a selective autophagy process, lipophagy can specifically recognize and encapsulate lipid droplets and transport them to lysosomes for degradation (26). Under the mediation of ALP, lipid droplets are decomposed into FFAs. On the one hand, these FFAs can be oxidized through the mitochondrial *β*-oxidation pathway, on the other hand, they can also be re-esterified or secreted out of the liver with very low density lipoprotein, thus effectively blocking the excessive accumulation of pathological lipids in the liver (27). A large number of genetic evidence supports this view: after knocking out the autophagy key gene Atg5 or Atg7 in mice, the basic autophagy flow is blocked, and the function of autophagy is significantly weakened, resulting in a large accumulation of lipid droplets in hepatocytes and spontaneous development into obvious hepatic steatosis, which fully reveals the indispensable protective role of ALP-mediated autophagy in maintaining liver lipid homeostasis (28). In addition, the function of ALP is not limited to the metabolic regulation of triglycerides, but also actively participates in the degradation and efflux of cholesterol in hepatocytes. By promoting the hydrolysis of cholesterol esters and the lysosomal clearance of free cholesterol, ALP can significantly reduce the abnormal deposition of cholesterol in hepatocytes, thereby alleviating pathological changes such as hepatocyte injury, inflammatory response and cholestasis caused by cholesterol overload. It has important physiological significance for the prevention and treatment of cholesterol-related liver diseases (29).

### 3.2 Maintenance of hepatic mitochondrial function

In addition to regulating lipid metabolism, ALP also plays a key role in maintaining liver mitochondrial homeostasis through mitophagy, a selective autophagy process, thereby effectively reducing the production of reactive oxygen species (ROS) and protecting liver cell function. Mitochondria is one of the most abundant organelles in hepatocytes. It not only provides a large amount of ATP for cells through oxidative phosphorylation, but also participates in important metabolic pathways such as fatty acid *β*-oxidation, urea synthesis and gluconeogenesis. It is the cornerstone of the liver to perform high-load energy conversion and substance synthesis (30). However, under the conditions of metabolic stress, exposure to toxic substances or aging, mitochondria are prone to damage, and the dysfunction of its electron transport chain can lead to electron leakage, which in turn produces excessive ROS. When ROS production exceeds the scavenging capacity of the antioxidant system, it will induce oxidative stress, attack lipids, proteins and DNA, eventually cause mitochondrial permeability transition, cytochrome c release, and even initiate apoptosis or necrosis signals, seriously damaging liver cell activity (31).

Mitophagy is a selective autophagy pathway in ALP and an important defense mechanism for cells to cope with this threat. It can specifically recognize and encapsulate damaged mitochondria, and target them to lysosomes for degradation and clearance, thereby cutting off the source of continuous production of ROS and effectively inhibiting the vicious cycle of oxidative damage (32). A large number of studies have shown that this process depends on the PINK1-Parkin pathway or other receptor-mediated recognition mechanisms. When the mitochondrial membrane potential is lost, PINK1 stably accumulates and recruits Parkin, and then ubiquitinates the mitochondrial outer membrane protein and initiates the capture of mitochondria by autophagy receptors. In animal experiments, in a mouse model with impaired mitophagy, a large number of damaged mitochondria accumulated in hepatocytes, accompanied by a significant increase in ROS levels, an increase in hepatocyte apoptosis index, and a significant increase in sensitivity to liver injury stimuli such as drugs or ischemia-reperfusion, suggesting that mitophagy defects are an important factor in susceptibility to liver injury. On the contrary, activation of mitophagy by pharmacological or genetic means can accelerate the removal of dysfunctional mitochondria, reduce intracellular ROS load, and reduce mitochondrial-dependent apoptosis signals, thereby significantly alleviating liver cell damage and improving liver function indicators (33). In addition, the control of mitochondrial quality by ALP is not solely dependent on the clearance process, but is closely coordinated with mitochondrial biosynthesis to jointly regulate the number, morphology and functional activity of mitochondria. When the metabolic demand increases, ALP can promote the formation of new mitochondria by regulating transcription co-activators such as PGC-1*α* while removing old mitochondria, so as to ensure the dynamic balance between energy supply and metabolic adaptability. This coupling mechanism of clearance and synthesis enables hepatocytes to flexibly adjust the mitochondrial network according to nutritional status and stress signals, which not only avoids the harmful accumulation of damaged mitochondria, but also meets the energy and metabolic needs of the liver under different physiological or pathological conditions (34). Therefore, ALP-mediated mitophagy and its synergistic effect with biosynthesis constitute the core hub of liver mitochondrial quality control, which is of irreplaceable significance for maintaining the overall metabolic homeostasis and anti-injury ability of the liver.

### 3.3 Participation in hepatic detoxification and injury repair

ALP also plays an important role in liver detoxification and tissue repair. By degrading abnormal proteins, toxic substances and damaged organelles, ALP maintains intracellular homeostasis and promotes the regeneration of hepatocytes after injury. The liver is the body's main detoxification organ, responsible for the metabolism of a variety of exogenous toxins (such as drugs, environmental toxins) and endogenous waste (such as misfolded protein). ALP can encapsulate these harmful substances and transport them to lysosomes for degradation into non-toxic or low-toxic products, which is convenient for subsequent excretion, thus reducing the burden of hepatocyte toxicity (35). When hepatocytes are damaged by oxidative stress or inflammation, ALP is activated to remove damaged mitochondria, endoplasmic reticulum fragments and denatured proteins in a timely manner, while recycling degradation products to provide energy and raw materials for cell repair. A large number of studies have confirmed that activation of ALP can significantly alleviate drug-induced liver injury, ischemia-reperfusion injury and other types of liver injury, reduce tissue necrosis and accelerate recovery. The defect of ALP function will aggravate the damage and hinder the repair process (36, 37). It is noteworthy that in MASLD, lipotoxicity can up-regulate the expression of METTL3 gene, resulting in an increase in the methylation level of Rubicon mRNA, which in turn leads to the accumulation of Rubicon protein. Rubicon is a negative regulator of autophagosome-lysosomal fusion. Overexpression of Rubicon blocks autophagic flux, inhibits lipid droplet clearance, and ultimately promotes the progression of MASLD (38). This mechanism reveals the important association between epigenetic modification and autophagy regulation, and also provides a potential target for the intervention of MASLD.

## 4 Core molecular mechanisms of ALP dysfunction in MASLD

The root cause of ALP dysfunction in MASLD is lysosomal dysfunction, accompanied by autophagosome-lysosomal fusion disorders, core regulatory factors abnormalities, and selective autophagy regulation disorders. These interrelated abnormalities jointly destroy ALP homeostasis and promote disease progression.

### 4.1 Lysosomal dysfunction

Lysosomal dysfunction is the core mechanism of impaired ALP function in MASLD, which is mainly manifested by insufficient lysosomal acidification, impaired lysosomal membrane integrity, and decreased lysosomal enzyme activity—among which acidification dysfunction is the most serious abnormality (39). The process of lysosomal acidification depends on the activity of V-ATPase. In MASLD hepatocytes, excessive free fatty acids inhibit the expression and activity of V-ATPase, thereby reducing the transport of H+ into the lysosomal lumen, resulting in insufficient acidification (23) (Figure 2).

**Figure 2:**
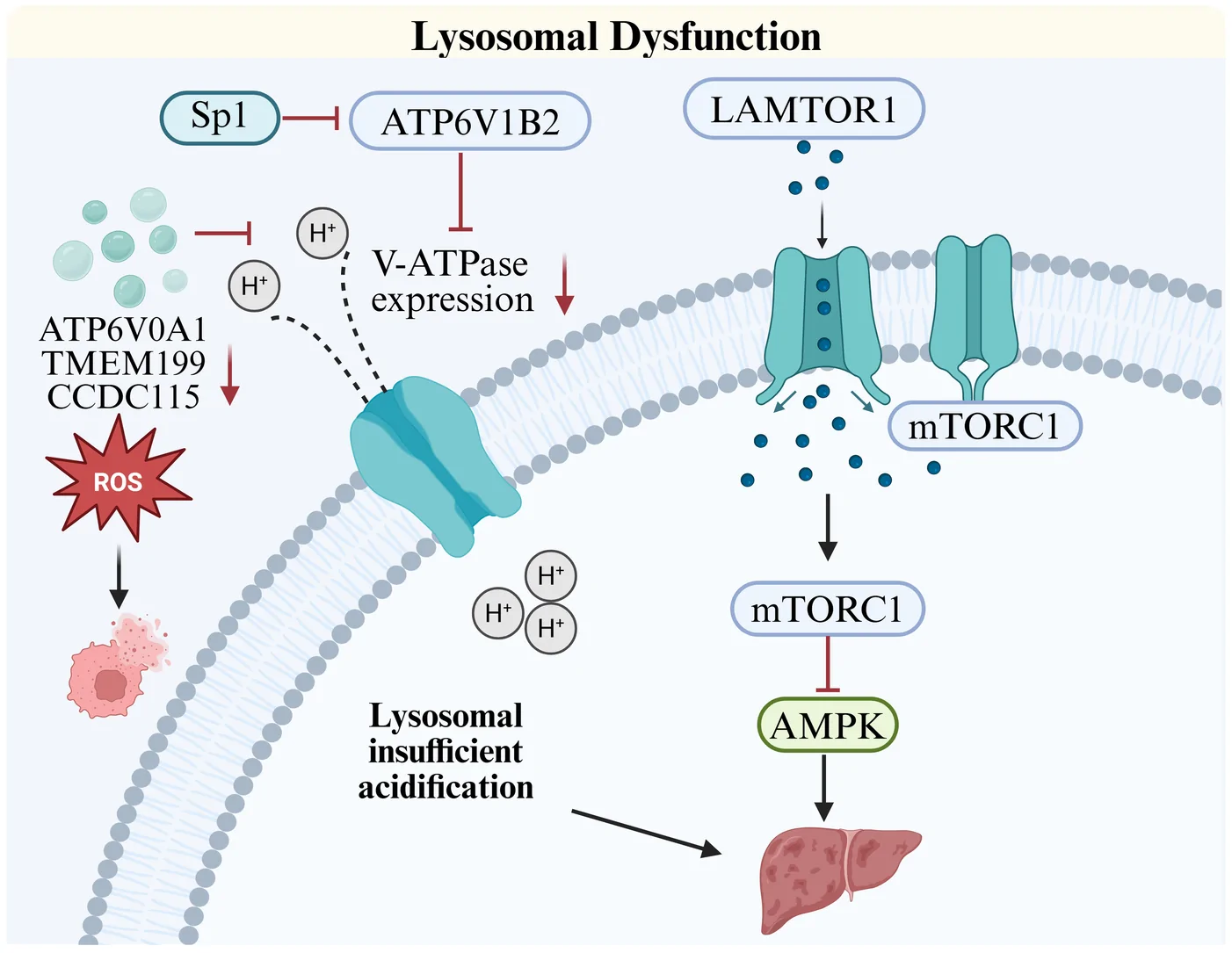
Mechanisms of lysosomal dysfunction in liver injury. Sp1 represses ATP6V1B2, while deletion of ATP6V0A1, TMEM199 and CCDC115 impairs V-ATPase-mediated lysosomal acidification. LAMTOR1 overactivates mTORC1 and thereby inhibits AMPK. Excessive ROS disrupts lysosomal membrane integrity. Insufficient lysosomal acidification, dysregulated mTORC1/AMPK signaling and accumulated ROS collectively induce lysosomal dysfunction, ultimately leading to hepatic pathological injury. Created in BioRender. Cheng, X. (2026) https://BioRender.com/5s3z0y6

Importantly, recent studies have gone beyond the general description of V-ATPase dysfunction and identified specific subunits as key regulatory targets. As a key subunit of V-ATPase, the expression level of ATP6V1B2 in the serum of MASLD patients was significantly decreased. Mechanistically, ATP6V1B2 can maintain the lysosomal acidification environment and promote the lysosomal degradation of fatty acid synthase (FASN), thereby inhibiting liver lipid accumulation. Knockdown of ATP6V1B2 aggravated lipid deposition in hepatocytes, inhibited lysosomal acidification and increased lysosomal membrane permeability, whereas its overexpression alleviated lipid deposition. Transcription factor Sp1 regulates the expression of ATP6V1B2-Silencing Sp1 can significantly increase the mRNA and protein levels of ATP6V1B2, indicating that there is a transcriptional regulator*y* axis that can be used for therapeutic intervention (8). Furthermore, cadmium exposure impairs lysosomal acidification and autophagic flux by promoting proteasome and ALP-mediated degradation of ATP6V0A1, another key V-ATPase subunit, leading to liver triglyceride accumulation, indicating that ATP6V0A1 is a key regulatory target in chemical fatty liver disease (40). The deficiency of V-ATPase assembly factors TMEM199 and CCDC115 can lead to impaired lysosomal acidification, dysfunction of lipophagy, abnormal accumulation of lipid droplets, and increased release of apolipoprotein B (apoB) -containing lipoproteins by hepatocytes, leading to hypercholesterolemia and hepatic steatosis (41). In the MASLD mouse model induced by high-fat diet (HFD), the expression of liver V-ATPase was significantly decreased, the lysosomal pH value was increased, and the acidification ability was significantly weakened. It is worth noting that the administration of V-ATPase activator can restore lysosomal acidification, improve ALP activity, and alleviate hepatic steatosis and inflammatory response (42).

In addition to acidification defects, excessive ROS accumulation in MASLD destroys lysosomal membrane integrity, resulting in leakage of lysosomal enzymes into the cytoplasm. This not only weakens the lysosomal degradation ability, but also causes abnormal cytoplasmic protein degradation, further exacerbating liver cell damage (43). The lysosomal gating protein LAMTOR1 (late endosomal/lysosomal adaptor protein, MAPK and mTOR activator 1) is another key regulatory node. LAMTOR1 is a lysosomal membrane adaptor protein that anchors mTORC1 to the surface of lysosomes (44). Recent studies have confirmed that LAMTOR1 is a direct molecular target of the natural compound Aca (ACA) by drug affinity response target stability (DARTS) technology combined with LC-MS/MS proteomics analysis: ACA binds to LAMTOR1 and releases it from the LAMTOR complex, thereby inhibiting MTORC1 and activating AMPK, thereby enhancing autophagy and improving MAFLD in the CDAHFD mouse model of rapid-onset steatohepatitis. LAMTOR1 gene knockdown can simulate the effect of ACA treatment, further confirming that it is a key therapeutic target (10). Therefore, the LAMTOR1-MTORC1-AMPK signaling pathway represents a unique lysosomal-centered ALP regulation pathway, which is different from the classical nutrient sensing signaling pathway.

The decrease of lysosomal enzyme activity is another important feature, and the decrease of LAL activity is particularly significant: LAL is responsible for the degradation of lipid substrates transported by autophagosomes; loss of its function impairs lipid degradation, promotes lipid droplet accumulation, and activates inflammatory signaling pathways, thereby aggravating liver inflammation (45, 46).

### 4.2 Impaired autophagosome-lysosome fusion

Autophagosome-lysosome fusion disorder is one of the core links of ALP dysfunction in MASLD.Once the fusion step is blocked, it will directly lead to the interruption of autophagic flow, so that substrates such as lipid droplets, damaged mitochondria and abnormal proteins cannot be effectively removed, thus accelerating liver cell damage and disease progression (Figure 3). The molecular basis of this fusion damage is mainly due to the abnormal expression or post-translational modification of key regulatory proteins, in which SNARE protein family members and Rab family small GTPases play a central role. Rab7, as an important molecule regulating late endocytosis and autophagosome maturation, is responsible for mediating the transport of autophagosomes along microtubules to lysosomes and promoting their docking, while Syntaxin 17 is a Q-SNARE protein located on the autophagosome membrane and directly involved in the formation of fusion pores (47). The study found that the expression level of Rab7 was significantly down-regulated in hepatocytes of MASLD patients and mouse models, accompanied by impaired STING (interferon gene stimulator) degradation pathway. STING is usually cleared through the autophagy-lysosomal pathway to avoid continuous activation of the inflammatory response, while the reduction of Rab7 leads to the inability of STING to be effectively degraded, which in turn aggravates the liver inflammatory microenvironment. These two aspects together hinder the effective fusion of autophagosomes and lysosomes, resulting in a large accumulation of autophagic substrates (48). At the same time, the decreased expression of Syntaxin 17 is also critical, because it must be co-assembled into a SNARE complex with VAMP8 on the lysosomal membrane and SNAP29 in the cytoplasm to close the two membranes and induce fusion. When Syntaxin 17 is insufficient, the formation of this functional SNARE complex is severely limited, the membrane fusion efficiency is significantly reduced, and the autophagosome is thus retained in the cytoplasm and cannot complete the degradation process (49). In addition, exogenous factors such as saturated fatty acids (such as palmitic acid) can disrupt intracellular calcium homeostasis and activate unfolded protein response by inducing endoplasmic reticulum stress, thereby specifically inhibiting autophagosome-lysosomal fusion steps. Even if autophagosome formation is normal, subsequent fusion events are still blocked, resulting in autophagic flux disorders and substrate accumulation, which is an important way for dietary factors to directly interfere with ALP function (50). The decrease of lysosomal acidification is also an indirect but important factor of fusion damage. The appropriate acidic pH environment (about 4.5–5.0) is not only a prerequisite for the maturation and activation of various hydrolases (such as cathepsins), but also directly regulates the conformation and assembly efficiency of the capture complexes on the membrane. If the acidification is insufficient, the capture complex cannot be formed correctly, the autophagosome cannot be docked stably, and the fusion process is thus damaged (51). In addition, the lysosomal membrane protein Sidt2 is also indispensable in fusion regulation. Studies have shown that the levels of p62 and LC3-II in Sidt2-knockout hepatocytes were significantly increased, indicating that a large number of autophagosomes were accumulated without degradation, and the content of mature cathepsin B was decreased, suggesting lysosomal dysfunction. This state blocks autophagosome-lysosomal fusion, which in turn weakens the efficiency of fatty acid *β*-oxidation, leading to abnormal accumulation of lipid droplets in hepatocytes and accelerating the progression of MASLD (52). In summary, the impaired autophagosome-lysosomal fusion involves multiple molecular events, from the decreased expression of Rab7 and Syntaxin 17, to the assembly disorder of SNARE complex, to fatty acid-induced endoplasmic reticulum stress and acidification defects, and the loss of Sidt2 function. These factors are intertwined and constitute the core mechanism of ALP dysfunction, and provide multiple potential targets for the intervention of MASLD.

**Figure 3:**
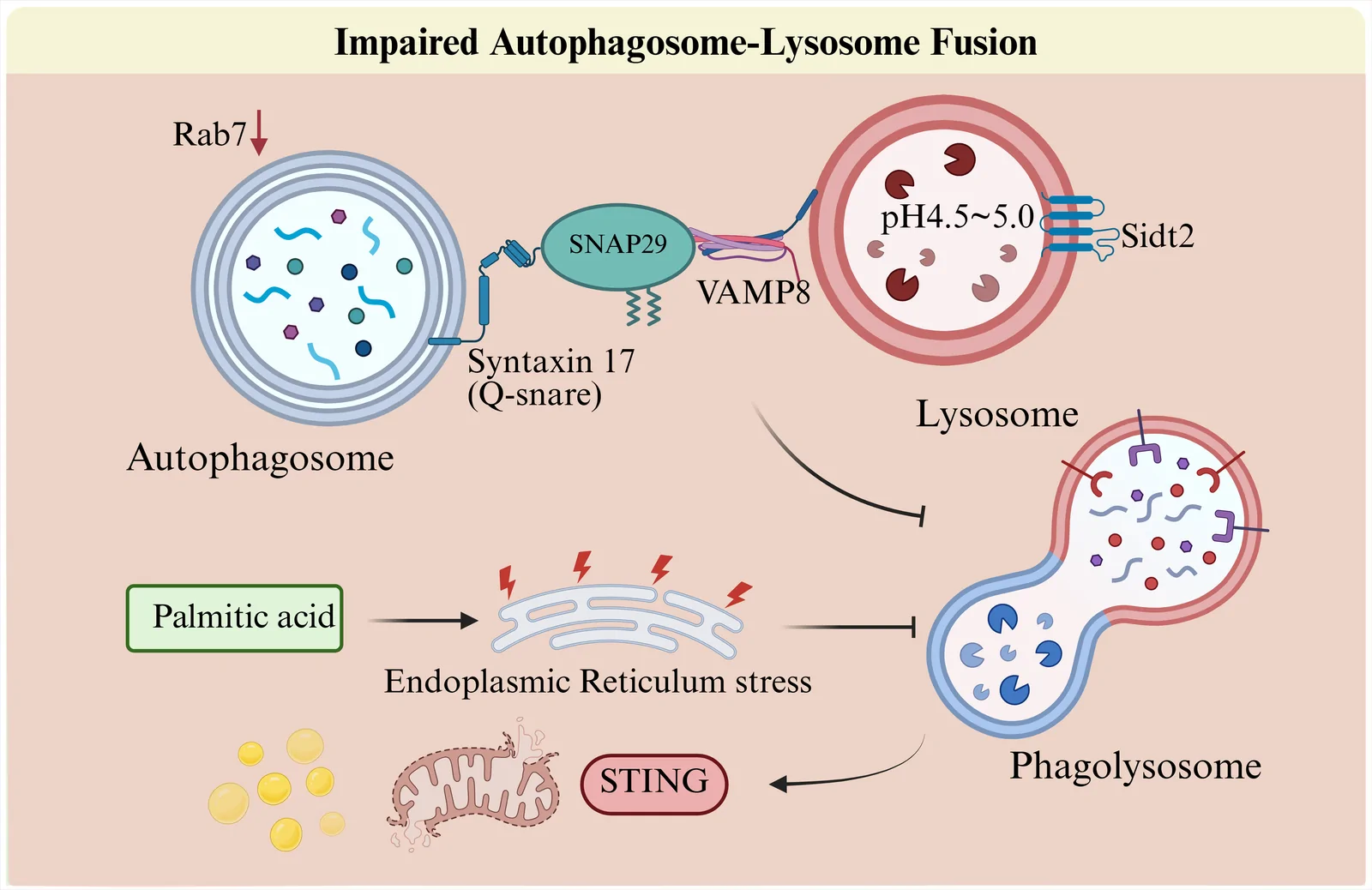
Molecular mechanisms underlying impaired autophagosome-lysosome fusion. Reduced Syntaxin17 expression disrupts assembly of the Syntaxin17-SNAP29-VAMP8 SNARE complex. Palmitic acid induces endoplasmic reticulum stress and downregulates Rab7 protein. Impaired autolysosome formation leads to accumulation of undegraded cargos, ultimately triggering hepatic lipotoxic injury. Created in BioRender. Cheng, X. (2026) https://BioRender.com/o72kqtq

### 4.3 Abnormalities in core regulatory factors

The imbalance of core regulatory factors is the main driving factor of ALP dysfunction in MASLD, mainly manifested as abnormal expression or activity of TFEB, AMPK and mTOR. TFEB is the main regulator of ALP. TFEB is dephosphorylated and transported to the nucleus in the absence of nutrients or lysosomal dysfunction, where it binds to gene promoters involved in autophagy and lysosomal biogenesis (including LC3, p62, LAL and V-ATPase subunits), thereby activating transcription of these genes and restoring autophagic flux (53) (Figure 4).

**Figure 4:**
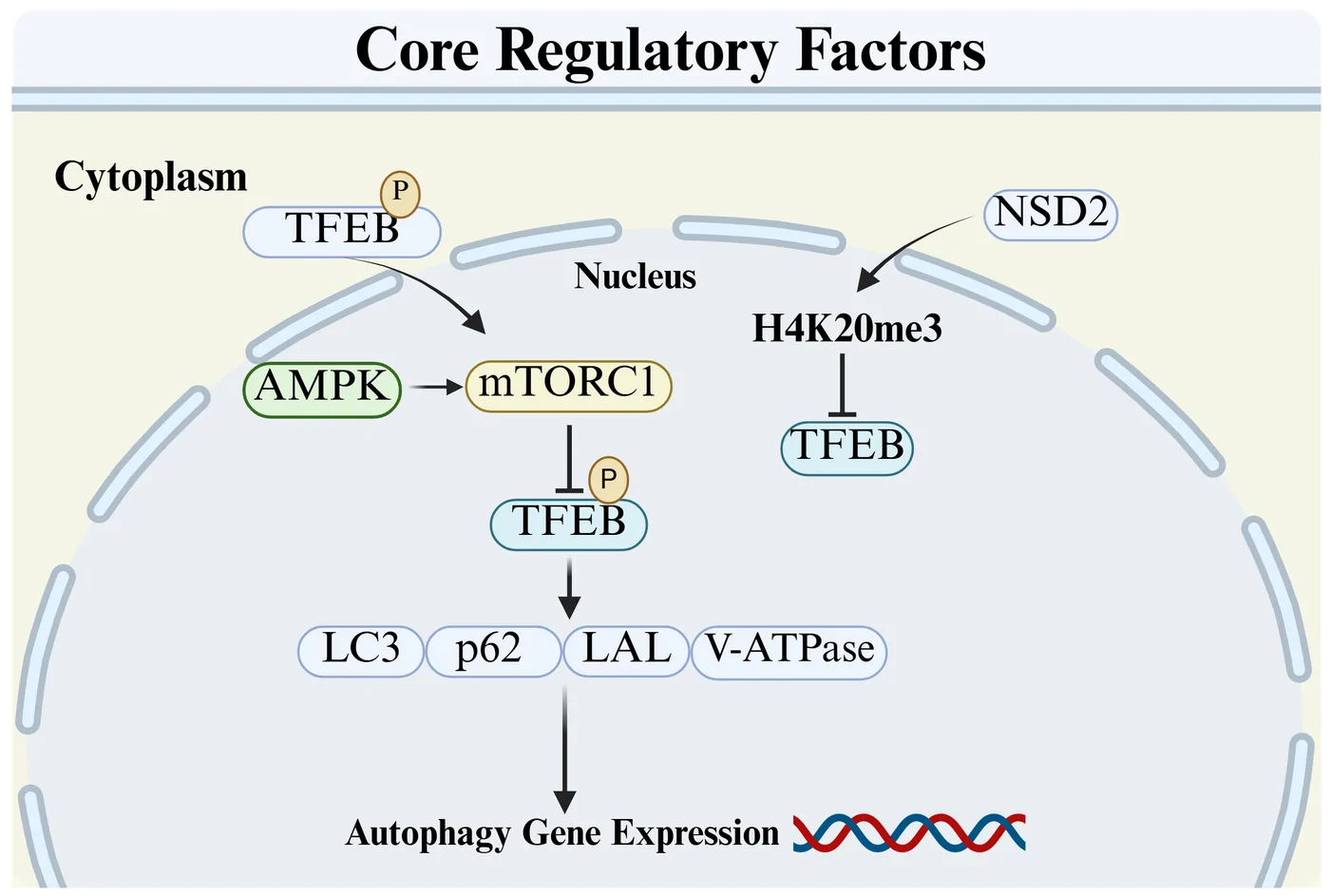
Core factors governing autophagic transcription. Cytoplasmic AMPK represses intranuclear mTORC1, which phosphorylates and inhibits TFEB activity. Epigenetically, NSD2 catalyzes H4K20me3 modification to suppress TFEB transcription. Activated TFEB initiates the expression of LC3, p62, LAL and V-ATPase, thereby coordinately upregulating the autophagy-associated gene program. Created in BioRender. Cheng, X. (2026) https://BioRender.com/rthxnkj

As the main transcriptional regulator of ALP, the activity of TFEB in MASLD is inhibited due to the excessive activation of mTORC1: mTORC1 phosphorylates TFEB and isolates it from the cytoplasm, thereby preventing its transport to the nucleus and subsequent transcriptional activation of ALP-related genes (54). In hepatocytes of MASLD patients and mouse models, mTORC1 activity was significantly increased, TFEB phosphorylation level was increased, and its transfer to the nucleus was reduced. These changes together lead to impaired ALP function. It is worth noting that drug inhibition of mTORC1 or overexpression of TFEB can activate ALP activity and improve fatty liver and inflammatory symptoms (55). As a key autophagy activator, the activity of AMPK in MASLD is weakened, resulting in the failure of its inhibitory effect on mTORC1, thereby blocking autophagy activation (56). Consistently, in a HFD-induced MASLD mouse model, AMPK activity was significantly reduced, and activation of AMPK promoted autophagy and alleviated liver lipid accumulation (57). Recent studies have revealed another mechanism of epigenetic regulation. Histone methyltransferase NSD2 has been identified as a key inhibitor of TFEB in MASLD. NSD2 catalyzes histone H4K20 trimethylation (H4K20me3) at the Tfeb locus, directly inhibiting TFEB transcription, thereby impairing the function of the entire ALP pathway. The expression of NSD2 at mRNA and protein levels was significantly increased in MASLD patients and HFD-fed mouse models, and was positively correlated with serum triglyceride, low-density lipoprotein, transaminase levels and liver pathology SAF score (9).

### 4.4 Dysregulation of selective autophagy

Selective autophagy gives substrate degradation process specificity, and its abnormal regulation is an important manifestation of MASLD autophagy-lysosomal pathway damage. The disorder of lipid autophagy, mitophagy and endoplasmic reticulum autophagy can aggravate liver cell damage and accelerate disease progression (58) (Figure 5).

**Figure 5:**
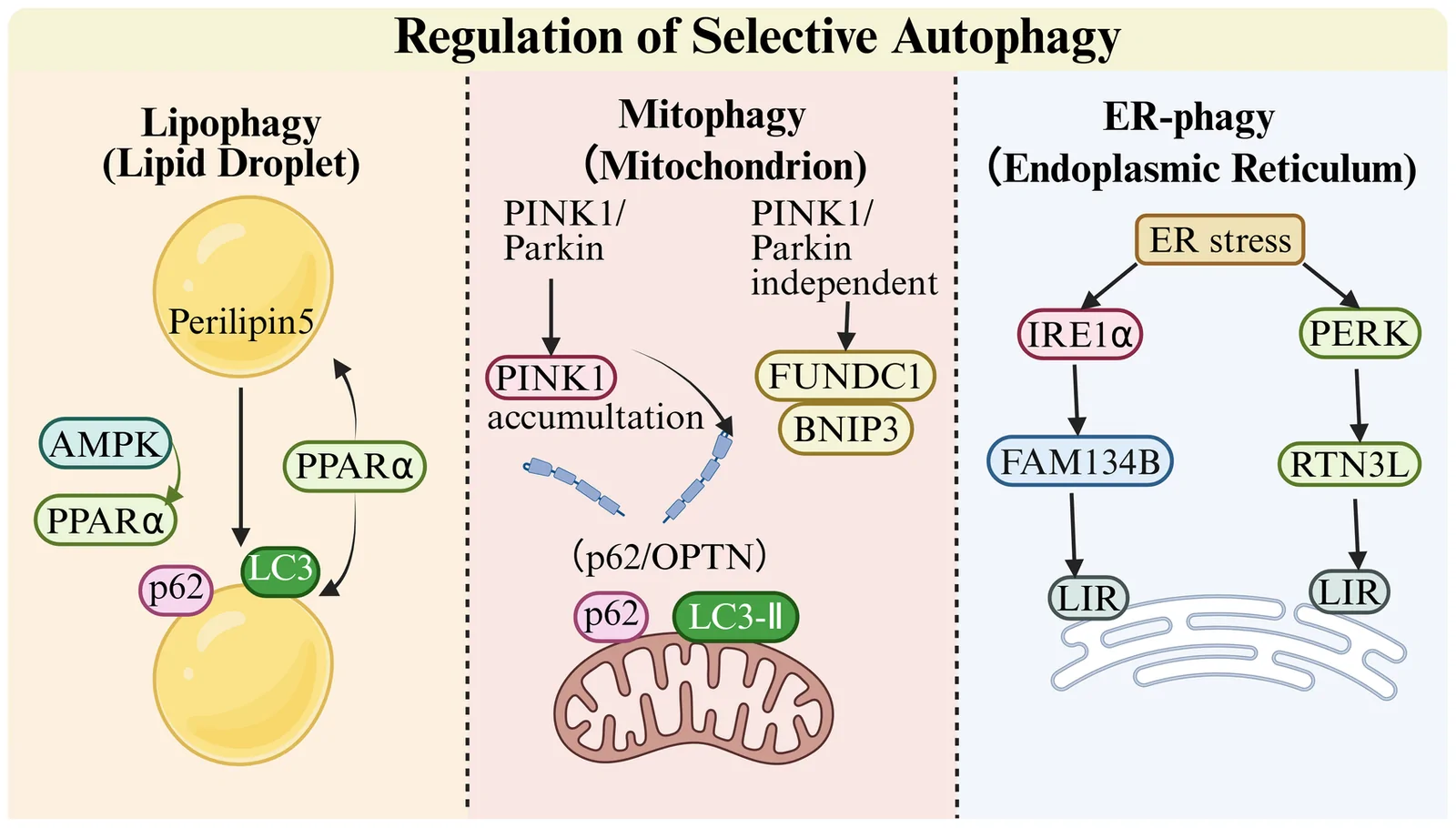
Regulatory mechanisms of three major subtypes of selective autophagy. In lipophagy, AMPK and PPAR*α* upregulate Perilipin5 to mediate the engulfment of lipid droplets via the p62-LC3 complex. Mitophagy operates through two pathways: the PINK1/Parkin-dependent pathway and the FUNDC1/BNIP3-independent pathway. Endoplasmic reticulum stress activates the IRE1*α*-FAM134B and PERK-RTN3L cascades to initiate ER-phagy relying on LIR motifs. Created in BioRender. Cheng, X. (2026) https://BioRender.com/u3x0aac

Lipophagy is regulated by lipid droplet-associated proteins (such as perilipin 5, which directly binds to LC3) and p62 protein, which is ubiquitinated to label lipid droplet proteins for autophagy recognition. AMPK and PPAR*α* signaling pathways enhance lipophagy by up-regulating related genes (59–61). In the early stage of MASLD, lipophagy is activated as a compensatory mechanism; however, in the MASH stage and the later stage, its activity is insufficient, and lipid droplets cannot be effectively degraded, resulting in gradual accumulation of lipids. This defect is closely related to the abnormal ubiquitination of lipid phagocytic receptors (such as p62) and the inhibition of PPAR*α* pathway (62). P62/SQSTM-1 is a key factor regulating autophagosome-encapsulated lipid droplets and their subsequent degradation by lysosomal hydrolase. Therefore, p62 agonists become a new strategy for the treatment of hepatic steatosis by promoting autophagy-mediated lipid droplet clearance (63).

Mitophagy is mainly mediated by the PINK1/Parkin pathway: damaged mitochondria accumulate PINK1, which recruits and activates Parkin, resulting in ubiquitination of mitochondrial outer membrane proteins; these ubiquitinated targets are then recognized by p62 or optineurin (OPTN) and transported to autophagosomes (64). In addition, mitophagy receptors such as FUNDC1 and BNIP3 can directly bind to LC3 without relying on the PINK1/Parkin pathway (65, 66). In MASLD, mitophagy is insufficient to clear damaged mitochondria, leading to excessive accumulation of ROS and increased oxidative stress damage (67). In the MASH mouse model, the activity of PINK1/Parkin pathway and the expression of mitophagy receptors were significantly reduced, resulting in the accumulation of damaged mitochondria. Activation of mitophagy reduces ROS production and alleviates liver cell damage (68).

Endoplasmic reticulophagy (ER-phagy) is dependent on endoplasmic reticulum resident receptors (such as FAM134B, RTN3L) containing LC3 interaction domain (LIR), which bind to LC3 and recruit damaged endoplasmic reticulum fragments to autophagosomes. Endoplasmic reticulum stress can also further activate ER-phagy through IRE1*α* and PERK signaling pathways as an adaptive protection mechanism to alleviate stress (69, 70). In MASLD, the activation of ER-phagy is insufficient to degrade the damaged endoplasmic reticulum, thereby prolonging endoplasmic reticulum stress and exacerbating liver cell damage (71). Although the endoplasmic reticulum stress pathway is activated in MASLD, the expression of ER-phagy receptor FAM134B is significantly reduced, resulting in insufficient activation of ER-phagy activation and failure to alleviate ER stress (72).

### 4.5 Crosstalk between ALP dysfunction and ferroptosis

Ferroptosis is a form of regulated cell death driven by lipid peroxidation and dependent on iron ions. In recent years, it has been confirmed as a key pathogenic mechanism throughout the MASLD spectrum. In view of the central role of the liver in iron metabolism and lipid metabolism, hepatocytes are highly susceptible to ferroptosis, which exacerbates liver oxidative stress, inflammation and fibrosis (73). Importantly, ferroptosis has been proposed as a “central hub” connecting iron metabolism disorders, abnormal lipid accumulation and oxidative stress imbalance, and plays a key role in the whole process from benign steatosis to inflammatory infiltration, fibrosis and even HCC development (74).

The synergistic effect of ALP dysfunction and ferroptosis constitutes a key pathogenic mechanism. ALP dysfunction can lead to the accumulation of damaged mitochondria and lipid droplets, which are the substrate sources of lipid peroxidation. When the autophagic clearance function is impaired, lipid peroxides accumulate unrestrainedly, leading to inactivation of glutathione peroxidase 4 (GPX4) and ferroptosis (75) (Figure 6). Experimental studies have shown that early life intake of HFD can induce prepubertal obesity-related MASLD through autophagy-mediated ferroptosis pathway, which indicates that the ALP-ferroptosis axis has been activated in the early stage of the disease (76).

**Figure 6:**
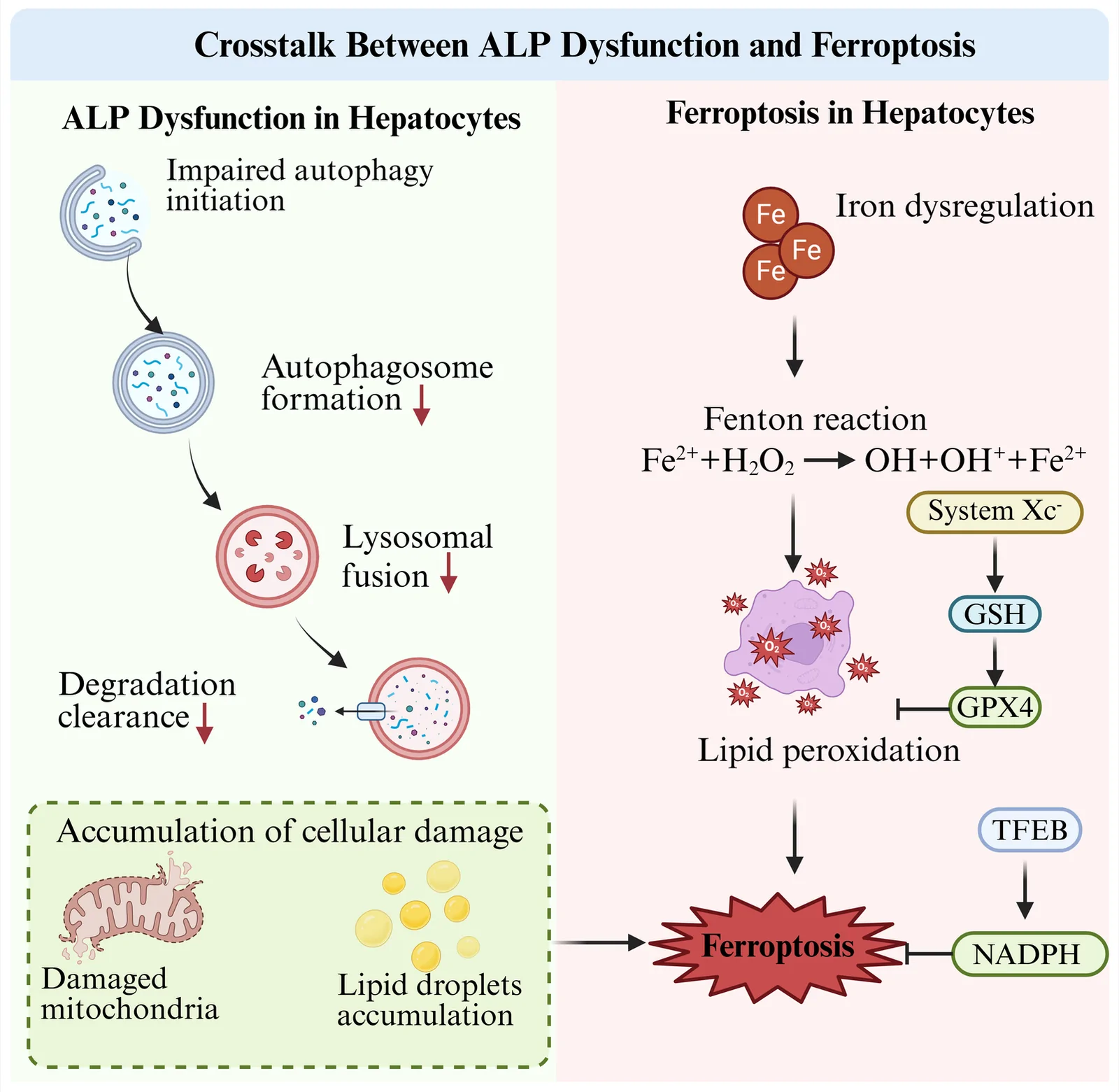
Crosstalk regulatory mechanism between ALP dysfunction and ferroptosis in hepatocytes. Multiple steps of ALP are blocked, including autophagy initiation, autophagosome biogenesis, lysosomal fusion and degradative clearance, resulting in accumulation of damaged mitochondria and lipid droplets and aggravated lipid peroxidation. Dysregulated System Xc-/GSH/GPX4 axis synergistically triggers hepatocyte ferroptosis. In contrast, TFEB in Kupffer cells confers resistance to ferroptosis by promoting NADPH production. Created in BioRender. Cheng, X. (2026) https://BioRender.com/u3x0aac

It is worth noting that the protective effect of TFEB in Kupffer cells may intersect with the regulation of ferroptosis. The induction of TFEB in Kupffer cells can enhance the production of NADPH through the pentose phosphate pathway, thereby enhancing the cell reduction ability to fight ferroptosis. This suggests that ALP dysfunction in non-parenchymal cells not only promotes disease progression by impairing lysosomal function, but also makes these cells more prone to ferroptosis (11). On the contrary, ferroptosis-related biomarkers (serum ferritin, transferrin saturation, malondialdehyde, oxidized phospholipids) are closely related to the severity of liver fibrosis and inflammation. Targeted intervention of ferroptosis with iron chelators, antioxidants or lipid peroxidation inhibitors can be used as a supplementary strategy for ALP treatment (12). Understanding this two-way interaction mechanism has far-reaching therapeutic significance for preventing the transformation of simple steatosis to MASH and advanced liver disease.

## 5 Dynamic functional changes of ALP at different stages of MASLD with cell-type specificity

The progression of MASLD represents a continuous pathological continuum during which the pathophysiological status of hepatocytes has undergone dramatic changes—from simple steatosis to advanced cirrhosis and HCC. Based on the molecular mechanisms described above, ALP function exhibits dynamic changes, shifting from compensatory activation to functional blockade, which eventually aggravates MASLD progression (77). Critically, the functional status of ALP varies not only across disease stages but also among different hepatic cell types, adding a layer of complexity to understanding its pathogenic roles (Figure 7).

**Figure 7:**
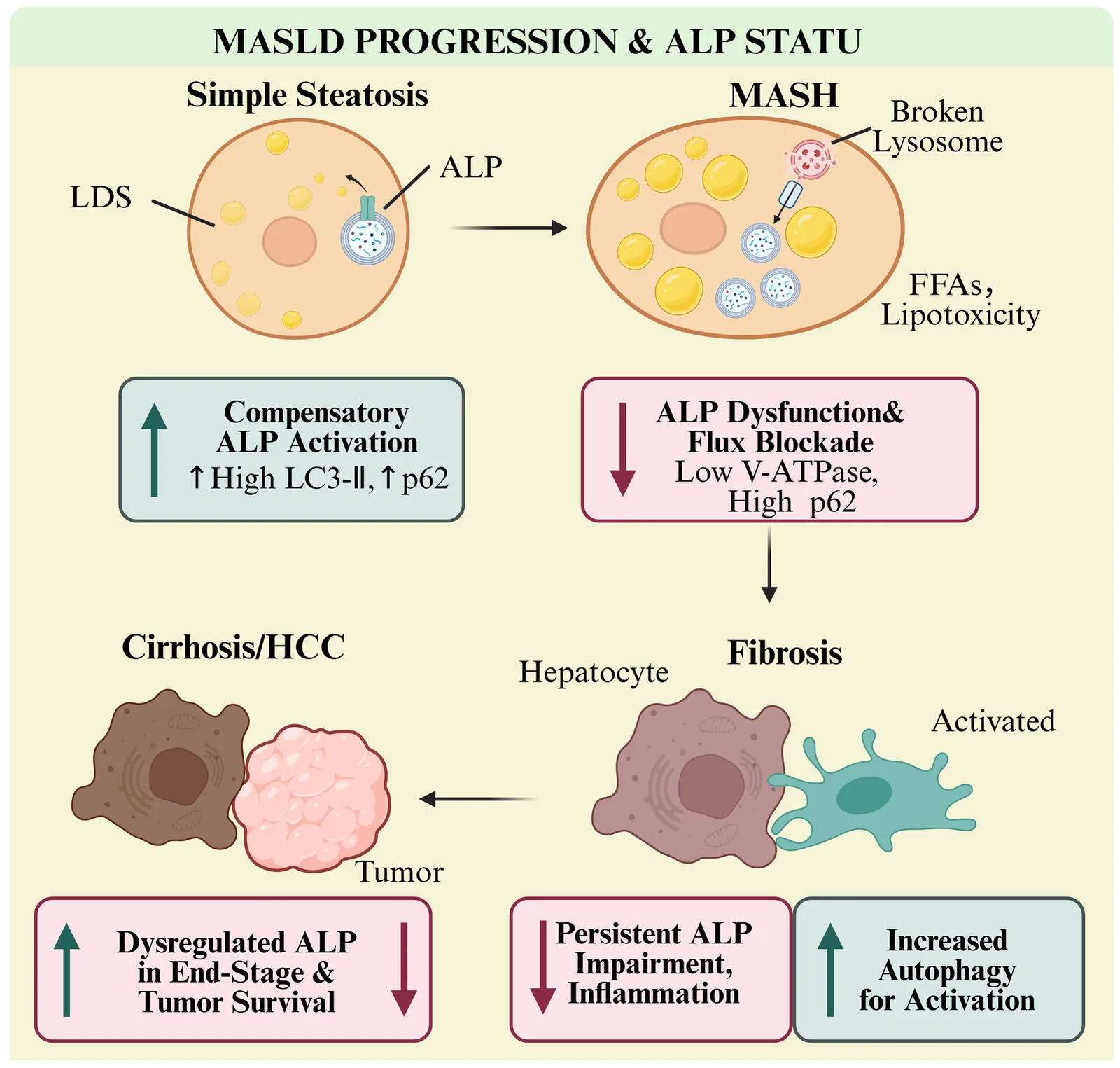
Dynamic alterations of the ALP throughout the progression of MASLD. ALP is compensatorily activated with elevated LC3-II and p62 levels in simple steatosis. During MASH, lysosomal rupture and accumulated lipotoxicity block ALP flux, and persistent injury induces hepatic fibrosis. ALP dysregulation facilitates hepatic stellate cell activation, while aberrantly regulated ALP sustains tumor cell survival in end-stage cirrhosis and HCC. Created in BioRender. Cheng, X. (2026) https://BioRender.com/qcn2a6p

### 5.1 Simple steatosis: compensatory activation

Simple steatosis represents the early stage of MASLD, which is characterized by the excessive accumulation of triglycerides in hepatocytes without hepatocyte damage or inflammation (78). At this stage, ALP is compensatorily activated, which is mainly manifested as enhanced lipophagy that degrades excessive lipid droplets to maintain hepatic lipid homeostasis (79). Studies have demonstrated that activated ALP is closely related to the increase in lipolysis of adipose tissue and isolated adipocytes of hyperketonemic dairy cows (80). In addition, in hepatocytes from patients with simple steatosis and in corresponding mouse models, the expression of LC3-II is significantly elevated while the expression of p62 decreased, indicating that autophagy activity was enhanced (81). Notably, autophagic flux displays a biphasic pattern, characterized by an initial activation followed by subsequent suppression, during the progression of hepatic steatosis induced by short-term vs. long-term HFD feeding (82). In the setting of mild fatty liver, the hepatic AMPK-TFEB-autophag*y* axis is activated, accompanied by inhibition of mTORC1. However, this protective response is lost as the disease advances to moderate fatty liver, which is associated with impaired AMPK/TFEB signaling and hyperactivation of mTORC1 (83). This compensatory activation represents the self-protective mechanism of hepatocytes, which can partially alleviate lipid-induced cellular injury. However, when the accumulation of lipids exceeds the degradation capacity of ALP, the compensation activation gradually fails, allowing the disease to progress to MASH (84).

### 5.2 MASH stage: autophagic flux blockade

MASH is a severe subtype of MASLD, which is histologically defined as hepatocyte steatosis, balloon degeneration, lobular inflammation and mild liver fibrosis (85). At this stage, the function of ALP is obviously impaired, and the interruption of autophagic flux is a key driver of disease progression (86). In hepatocytes from MASH patients and mouse models, extensive autophagosome accumulation, a significant decrease in LC3-II expression, and significantly elevated p62 expression have been observed, indicating autophagic flux blockade (87). Furthermore, the reduction of lysosomal acidification and the deep reduction of lysosomal hydrolase activity (such as LAL, CTSB) further confirmed ALP dysfunction. The core mechanism of this damage involves excessive FFAs within hepatocytes inhibiting the activity of V-ATPase on the lysosomal membrane, leading to insufficient lysosomal acidification, impaired autophagosome-lysosome fusion, and ultimately interrupting autophagic flux. This cascade results in massive accumulation of lipid droplets and abnormal proteins, causing lipotoxicity and inflammation, thereby exacerbating hepatocellular injury (88). As MASH progresses, persistent ALP dysfunction drives the transition to liver fibrosis, where a cell-type-specific functional imbalance emerges.

### 5.3 Liver fibrosis: cell-type-specific functional dichotomy

Liver fibrosis originates from the gradual evolution of MASH, which is characterized by hepatic stellate cell (HSC) activation, extensive collagen deposition, and fibrous scar formation (89). In the process of fibrosis, ALP exhibits a cell-type-specific functional dichotomy.

In hepatocytes, autophagic flux is often impaired due to lysosomal dysfunction, leading to the accumulation of lipid droplets, mitochondrial damage and the release of inflammatory mediators, which indirectly promotes the activation of HSC (90). In fibrotic patients and mouse models, the further accumulation of p62 and the persistent lipid toxicity in hepatocytes activated HSC through signaling pathways such as NF-*κ*B and TGF-*β*1 (91). In contrast, the autophagy activity is significantly enhanced in activated HSC, providing energy and biosynthetic precursors to maintain its long-term activation by degrading intracellular lipid droplets (92). Recent studies have elucidated the molecular mechanism of HSC autophagy. Liquid-liquid phase separation (LLPS), as a biophysical mechanism, can organize a variety of cellular processes including selective autophagy. RAB18 is a Ras-related small GTPase involved in lipid metabolism and has been shown to undergo phase separation in hepatic stellate cells (HSCs). During the progression of liver fibrosis, the phase separation of RAB18 promotes the formation of mitochondrial-associated membrane (MAM) structure, thereby accelerating the aggregation of RAB18. RAB18 in the phase separation state inhibits lipophagy by recruiting carnitine palmitoyltransferase 1A (CPT1A) and enhancing the succinylation reaction of dihydrolipoamide dehydrogenase (DLD), which ultimately leads to copper death in hepatic stellate cells (93). Natural compounds such as diallyl trisulfides (DATs) in garlic can bind to the K153 site of RAB18 and induce phase separation, thereby selectively protecting hepatocytes while activating the copper death process of hepatic stellate cells and exerting anti-fibrosis (94). Kupffer cells (KCs) are the liver's resident macrophages and play a key role in keeping the liver tissue balanced. During the course of MASLD, the composition of liver macrophages changes, and losing the original KCs is a hallmark of disease progression. In the HFHS diet and CDAA diet MASLD model, inducing TFEB expression in KCs can protect it from cell death. Maintaining KCs activity by TFEB induction can reduce liver steatosis by relying on macrophage lysosomal lipolysis and mitochondrial fatty acid oxidation. This protective effect stems from enhanced lysosomal function and increased NADPH production, thereby effectively combating oxidative stress and ferroptosis. KCs-specific TFEB overexpression represents a novel therapeutic strategy (11). In disease conditions, pathological remodeling in three different types of liver cells works together to speed up liver fibrosis: protective autophagy in liver cells is impaired, pro-fibrotic autophagy levels are increased in HSCs, and the survival and functional activity of Kupffer cells are suppressed.

Importantly, the above models based on cell type classification are increasingly supported by recent single-cell RNA sequencing (scRNA-seq) and spatial transcriptomics studies. Single-cell studies have begun to address the ALP in dispersed liver cell subsets, revealing a functional dichotomy that cannot be captured by bulk tissue analysis. Using scRNA-seq technology, Fredrickson et al. confirmed that vertical sleeve gastrectomy can induce a reparative macrophage state, in which the lysosomal activity of TREM2+ lipid-associated macrophages (LAMs) is enhanced. The loss of TREM2 eliminated the therapeutic benefits, indicating that this macrophage subset and its lysosomal regulatory program were necessary for MASH regression (95). Mechanistically, TREM2-dependent efferocytosis of apoptotic hepatocytes requires strong lysosomal degradation. Consistent with this, Ganguly et al. proved by scRNA-seq that TREM2+ macrophages are more efficient collagen-degrading cells, and their deletion hinders the elimination of steatosis and the inactivation of HSCs. Collagen degradation is also dependent on lysosomal cathepsins, which further supports the reparative role of ALP activity in TREM2+ LAMs (96). In contrast, Zheng et al. integrated single-cell data from human cirrhosis and found that autophagy was gradually inhibited as macrophages differentiated. Spatial analysis confirmed that autophagy in the fibrosis niche was weakened, and CXCR4- and S100A10-positive macrophages were enriched near the COL1A1-positive septum, indicating that the survival-reprogramming state near the septum may perpetuate fibrosis through impaired clearance (97). The ALP state in liver macrophages is subset-specific and disease-stage-dependent. TREM2+ LAMs represent a treatable state in which enhanced ALP promotes resolution, while autophagy-inhibited terminal macrophages in cirrhosis represent a dysfunctional endpoint that may lock in fibrotic remodeling. Liver cirrhosis and Hepatocellular Carcinoma Stage.

### 5.4 Liver cirrhosis and HCC are the terminal manifestations of MASLD

Liver cirrhosis is pathologically defined as normal liver structure replaced by fibrous scars, accompanied by severe liver dysfunction and portal hypertension. HCC is a malignant outcome of progressive cirrhosis, resulting from the accumulation of genetic and epigenetic changes in hepatocytes in the cirrhosis microenvironment (98). At this late stage, ALP dysfunction and abnormal autophagy synergistically drive disease progression through different mechanisms in different cell populations.

In liver cirrhosis, the continuous blockage of ALP and the continuous interruption of autophagic flux in hepatocytes aggravates hepatocyte damage and worsens liver function. The accumulation of p62/SQSTM1 caused by impaired autophagy clearance further activates NF-*κ*B and Nrf2 signaling pathways, continues pro-inflammatory and pro-oxidative states, and aggravates liver cell damage (91). At the same time, dysfunctional mitochondria and lipid droplets accumulate uncontrolled, amplifying oxidative stress and lipotoxicity. On the basis of this hepatocyte injury, hepatic stellate cells (HSCs) exhibit enhanced autophagy activity, providing fuel for their continuous activation, proliferation, and massive collagen deposition-a process that critically depends on autophagy-mediated lipophagy, providing energy and biosynthetic precursors for the metabolic needs of HSCs (92). The combination of impaired hepatocyte protection and continuous activation of HSC eventually leads to the formation of cirrhotic nodules and the deterioration of liver structure and function (99).

In HCC, ALP exhibits a paradoxical switch: during steatosis and MASH, ALP function is impaired in hepatocytes, but becomes abnormally activated in tumor cells. This reactivated autophagy maintains protein homeostasis and mitochondrial integrity by degrading abnormal proteins and damaged organelles, thereby supporting tumor cell survival, inhibiting apoptosis, and promoting proliferation, invasion and metastasis (100). Mechanistically, HCC cells up-regulate autophagy to cope with high metabolic needs and increased oxidative stress associated with rapid proliferation, forming a dependence on autophagic flux to gain survival advantages. Clinical studies have shown that compared with adjacent non-tumor tissues, the expression of LC3-II in HCC tumor tissues is significantly increased and the expression of p62 is decreased, indicating that autophagy activity is enhanced, and it is positively correlated with tumor grade, metastatic potential and poor prognosis of patients. Importantly, drug inhibition (such as chloroquine or hydroxychloroquine) or gene knockdown of essential autophagy genes (such as ATG5 or ATG7) can significantly inhibit HCC cell proliferation and invasion *in vitro* and in xenograft models, while promoting tumor cell apoptosis, suggesting that autophagy inhibition represents a promising therapeutic strategy for MASLD-related HCC (101). ALP activation is beneficial in the early stage of MASLD disease and harmful in HCC.This stage dependence emphasizes the importance of treatment timing and patient stratification, and highlights the stage-appropriate ALP regulation strategy that needs to reduce autophagy levels after malignant tumors.

## 6 Strategies and research progress of ALP-targeted therapy for MASLD

### 6.1 Pharmacological therapy targeting ALP

Small molecule drugs targeting ALP are an important research direction for the treatment of MASLD. These drugs mainly include ALP activators and lysosomal function regulators, which alleviate liver lipid accumulation and inflammatory response by enhancing ALP activity or restoring lysosomal function, thus opening up a new way for MASLD treatment (Table 1).

**Table 1:** Pharmacological therapy: targeting the autophagy-lysosomal system for MASLD.

Category	Intervention Substance/Drug	Developmental Stage / Investigational Models	Core Target & Regulated Pathway	Main Therapeutic Effects	References
ALP Activators	Rapamycin and its analogs (everolimus)	In vivo: HFD-induced MASLD mouse model	Inhibit mTORC1; regulate Perilipin 3 pathway	Activate ALP, enhance lipophagy, reduce hepatic lipid accumulation and inflammation in HFD-induced MASLD	(102, 103)
	Desloratadine, clomiphene citrate, liraglutide, exendin-4	In vitro: hepatocyte; In vivo: HFD-induced MASLD mouse model	Promote TFEB nuclear translocation; activate ALP-related gene transcription	Restore ALP function; ameliorate hepatic steatosis, inflammation and fibrosis	(29, 104–107)
	Irisin	In vitro: hepatocyte; In vivo: HFD-induced MASLD mouse model	Upregulate SIRT3; activate AMPK-mTOR-TFEB axis	Enhance autophagic flux; relieve hepatocyte lipid accumulation in MASLD	(108)
	Interleukin-24 (IL-24)	In vitro: hepatocyte; In vivo: HFFD-induced MASH mouse model	Bind IL-22R1/IL-20R2; activate AMPK-mTOR-TFEB axis	Restore autophagy-lysosome function; promote fatty acid oxidation; improve MASH	(109)
	NSD2 inhibitor (NSC663284)	In vitro: human liver organoid; In vivo: HFD-induced MASLD mouse model	Inhibit NSD2; relieve H4K20me3-mediated epigenetic suppression of TFEB	Restore TFEB transcription and ALP activity; counteract hepatic steatosis and disease progression	(9)
Lysosomal Function Modulators	ATP6V0A1	In vitro: hepatocyte; In vivo: cadmium-exposed / HFD mouse model	V-ATPase subunit; promote FASN lysosomal degradation	Alleviate hepatic steatosis by restoring lysosomal acidification	(8)
	Hepatic LAL	In vitro: hepatocyte; In vivo: ApoE-deficient mouse model	Upregulate liver X receptor α; modulate ALP activity	Improve cholesterol metabolism, hepatic steatosis and inflammatory fibrosis	(115)
	Sebelipase alfa	FDA-approved for LAL-D; NCT01371825 (original LAL-D trial)	Lysosomal acid lipase (LAL) replacement therapy	Supplement LAL deficiency; reduce hepatic lipid accumulation; MASLD application under investigation	(116, 117)
	Mitochondria-targeted AntiOxCIN_4_	In vitro: hepatocyte; In vivo: western-diet mouse model	Mediate PGC-1α-SIRT3 axis	Restore lysosomal proteolytic function; improve autophagic flux; mitigate MASLD	(118)
	CREBH	In vitro: hepatocyte; In vivo: HFD-induced MASH mouse model	Transcriptionally repress Coro1a (autophagosome-lysosome fusion inhibitor)	Restore autolysosome formation; alleviate MASH-associated hepatocyte injury, inflammation and fibrosis	(119)
Natural Products	Acacetin (ACA)	In vitro: hepatocyte; In vivo: CDAHFD-fed MASLD mouse model	Directly bind LAMTOR1→induce LAMTOR1 release from LAMTOR complex→MTORC1 inhibition→AMPK activation	Increase autophagy; ameliorate MAFLD in CDAHFD mouse model	(10)
	Nuciferine, polydatin	In vitro: hepatocyte; In vivo: HFD-fed MASLD/MASH mouse models	Inhibit lysosomal mTORC1; activate TFEB-mediated ALP	Attenuate hepatic steatosis, inflammation and lipid deposition in MASLD/MASH	(104, 120)
	Tetrahydrocurcumin	In vitro: hepatocyte; In vivo: HFD-fed MASH rat model	Inhibit mTORC1; induce TFEB nuclear translocation; upregulate lysosomal biogenesis	Alleviate NASH-related steatosis, inflammation and oxidative stress (reversed by mTORC1 activators)	(121)
	Buddleoside	In vitro: hepatocyte; In vivo: high-fat high-cholesterol-diet mouse model	Target PRKAB1; activate AMPK; inhibit mTORC1; activate TFEB-dependent ALP	Improve NASH in high-fat, high-cholesterol diet-fed mice	(122)
	Phillygenin	In vitro: hepatocyte; In vivo: MASLD cell-/mouse-models	Induce ER Ca²⁺ release; activate calcineurin; promote TFEB dephosphorylation/nuclear translocation	Restore autophagic flux/lysosomal biogenesis; enhance lipophagy; reduce hepatocyte lipid deposition	(123)
	Ajugol	In vitro: hepatocyte; In vivo: HFD-induced MASLD mouse model	Inhibit mTOR; promote TFEB nuclear translocation; enhance lysosomal biogenesis/fusion	Rescue impaired autophagic flux/lipophagy; ameliorate hepatic steatosis	(124)
	β-Sitosterol (Polygonum hydropiper-derived)	In vitro: hepatocyte; In vivo: CDAHFD / HFD-fed MASH mouse model	Regulate RAC1-mTOR axis; promote TFEB nuclear translocation	Repair lipophagy-lysosome defects; delay MASH progression	(125)
	Eucommia ulmoides leaf extract (EUL)	In vitro: hepatocyte; In vivo: HFD-fed rat model	Suppress mTOR-ER stress pathway	Correct MASLD lipid metabolism; enhance lysosomal function and autophagic flux	(126)
	Jiangzhi Granule	Small-sample clinical observation China; In vitro: hepatocyte; In vivo: HFD-induced MASLD mouse model	Regulate overall autophagy cascade	Promote autophagosome formation/fusion/degradation; protect mitochondria; relieve MASLD hepatocyte injury	(127)
	Huazhi Rougan Granule	In vitro: FFA-induced steatotic hepatocyte model	Activate SIRT1/AMPK pathway	Improve autophagic flux/lysosomal function; reduce free fatty acid-induced hepatocyte steatosis	(128)
Nano-drug Delivery Systems	Torin1-loaded ROS-responsive nanoparticles	In vitro: hepatocyte; In vivo: HFD mouse model	Target lysosomes; activate TFEB pathway	Enhance lysosomal function; facilitate lipid degradation; alleviate MASLD	(130)
	Autophagy inhibitor-loaded sequential delivery system	In vitro: HSC; In vivo: liver-fibrosis mouse model	Elevate autophagosomal/lysosomal pH; block autolysosome formation	Inhibit hepatic stellate cell activation; attenuate liver fibrosis	(129)
	Attapulgite	In vitro: hepatocyte; In vivo: HFD-induced MASLD mouse model	Target acidic lysosomes; restore lysosomal acidification; activate autophagic flux	Promote substrate degradation; exert hepatic protection in MASLD models	(110)
Gut-Liver Axis Modulation	Microbiota-derived H₂S (from Desulfovibrio etc.)	In vitro: hepatocyte; In vivo: HFD-induced MASLD mouse model	Induce c-Kit⁺ cDC1 autophagic cell death and liver inflammation	Link gut dysbiosis directly to ALP dysfunction in MASH	(87)
	Lactobacillus salivarius SNK-6-derived extracellular vesicles	In vitro: intestinal/hepatic cell; In vivo: HFD-fed MAFLD mouse model	Enhance hepatocyte mitophagy through Beclin-1/PPAR pathway; repair intestinal barrier; modulate gut microbiota composition	Improve systemic metabolic profiles; reduce hepatic lipid accumulation	(131)

#### 6.1.1 ALP activators

ALP activators enhance pathway function by promoting autophagy initiation or facilitating TFEB nuclear translocation. The two main classes are mTOR inhibitors and TFEB agonists. Rapamycin and its analogs (e.g., everolimus) inhibit mTORC1 activity, relieving its suppressive effects on the autophagy initiation complex and TFEB, thereby activating autophagy and ALP (102). In HFD-induced MASLD mouse models, rapamycin significantly activates ALP, enhances lipophagy, reduces hepatic lipid accumulation, and alleviates hepatic inflammation via the Perilipin 3 pathway (103). TFEB governs the expression of genes involved in autophagosome formation and lysosomal biogenesis. TFEB agonists, including desloratadine, clomiphene citrate, liraglutide, and exendin-4, directly promote TFEB nuclear translocation, activate ALP-related gene transcription, restore ALP function, and reduce hepatic steatosis, inflammation, and fibrosis (29, 104–107). Irisin is a protein hormone secreted by muscle induced by exercise. It has been found that it up-regulates SIRT3, activates AMPK-mTOR-TFEB axis, enhances autophagic flux, and alleviates lipid accumulation in HFD-induced MASLD (108). Similarly, interleukin-24 (IL-24) has also been found to bind to the IL-22R1/IL-20R2 complex to activate the AMPK-mTOR-TFEB axis, restore autophagy-lysosomal function, promote fatty acid oxidation, and ameliorating MASH (109).

In addition, because NSD2 inhibits TFEB transcription by catalyzing H4K20me3 modification, autophagy dysfunction and hepatic steatosis are induced. Liver cell-specific knockout of NSD2 increases LC3-II and decreases p62 in NSD2-deficient liver, and the activity of lipophagy and mitophagy is enhanced, which can enhance the body 's ability to resist high-fat-induced hepatic steatosis. Recovery of NSD2 expression will offset this protective effect (9). Therefore, the use of inhibitors such as NSC663284 to target NSD2 is a promising epigenetic treatment strategy for MASLD.

#### 6.1.2 Lysosomal function modulators

Lysosomal function modulators restore ALP activity by regulating lysosomal acidification and enhancing lysosomal enzyme function (110). Lysosomal acidification homeostasis, which determines degradative capacity and autophagic activity, is primarily regulated by V-ATPase, lysosomal ion channels, and lysosomal biogenesis pathways (111). Existing lysosomal acidification regulators fall into two categories: inhibitors and enhancers. Inhibitors of lysosomal acidificationincluding V-ATPase inhibitors (bafilomycin A1, concanamycin A), weak bases (chloroquine, hydroxychloroquine, ammonium chloride), and ionophores (monensin, nigericin), these agents are primarily used to block lysosomal acidification and autophagy (112–114). Enhancers improve acidification defects by activating V-ATPase, modulating TRPML1 or TMEM175, or activating the TFEB-mTOR pathway (111).

The identification of V-ATPase subunit-specific defects opens up new avenues for treatment. The expression of ATP6V1B2 in MASLD patients was significantly decreased, and the recovery of its expression could promote FASN lysosomal degradation and alleviate steatosis. In addition, targeting ATP6V0A1 (which is degraded in cadmium-related fatty liver) may become another subunit-specific treatment strategy (8). Hepatic LAL promotes cholesterol transport and bile excretion by up-regulating liver X receptor *α* and its downstream genes, regulating ALP function, thereby improving cholesterol metabolism disorders, hepatic steatosis and inflammatory fibrosis (115). Lysosomal enzyme replacement therapy is another important strategy: the replacement of LAL with Sberipase *α* can supplement the defective LAL in patients with MASLD, enhance lipid degradation and reduce liver lipid accumulation (116). Although this therapy has been approved for the treatment of liver diseases associated with LAL deficiency, its clinical application in MASLD needs further study (117). Mitochondrial targeted antioxidant AntiOxCIN_4_ can restore lysosomal proteolysis and improve autophagic flux through the PGC-1*α*-SIRT3 axis, thereby alleviating MASLD (118). Additionally, CREBH promotes autophagic lysosome formation and autophagic flux by transcriptionally inhibiting the negative regulator Coro1a of autophagosome-lysosome fusion, thereby reducing MASH-related hepatocyte injury, inflammation and fibrosis (119).

#### 6.1.3 Natural products

Natural products have become an ideal candidate for for MASLD therapy targeting ALP owing to their favorable safety characteristics and low adverse reactions. Recent studies have identified the specific molecular targets of many of these compounds.

Recently, the natural compound Acaoxetine has been proved to be a direct binding agent of LAMTOR1. Acaoxetine can promote the release of LAMTOR1 from the LAMTOR complex, inhibiting MTORC1 and activating AMPK, thereby enhancing autophagy and improving MAFLD (10). Similarly, by inhibiting the localization and activity of mTORC1 on lysosomes, nuciferine and polydatin activated TFEB-mediated ALP activity and reduced hepatic lipid accumulation, which significantly improved hepatic steatosis, inflammation and hepatocyte injury in MASLD/MASH mouse models (104, 120). Tetrahydrocurcumin can promote lysosomal biosynthesis by inhibiting mTORC1-induced TFEB translocation to the nucleus, and effectively improve liver steatosis, inflammation and oxidative stress in NASH rats. These effects can be eliminated by mTORC1 activator (121). Buddleoside activates AMPK pathway by targeting PRKAB1 subunit, thereby inhibiting mTORC1 and then activating TFEB-mediated ALP activity, effectively improving NASH in mice fed a high-fat and high-cholesterol diet (122). Phillygenin can induce the release of ER Ca^2+^ to activate calcineurin, promote the dephosphorylation and nuclear translocation of TFEB, restore autophagic flux and lysosomal biosynthesis, enhance lipophagy, and reduce lipid deposition and inflammatory response in hepatocytes in MASLD models (123). Ajugol, an active alkaloid isolated from Rehmannia glutinosa roots, can inhibit mTOR and promote TFEB nuclear translocation, enhance lysosomal biosynthesis and autophagosome-lysosomal fusion, thereby repairing damaged autophagic flux and lipophagy, and significantly alleviating hepatic steatosis (124). *β*-Sitosterol extracted from Polygonum hydropiper promotes TFEB nuclear translocation by regulating the RAC1-mTOR axis, repairs lipophagic-lysosomal dysfunction and delays the progression of MASH (125). Eucommia ulmoides leaf extract (EUL) regulates lipid metabolic disorders in MASLD models by inhibiting the mTOR-ER stress pathway, thereby improving lysosomal function and autophagic flux efficienc (126).

Traditional Chinese medicine formulations also show significant efficacy. Jiangzhi granules can promote the formation, fusion and degradation of autophagosomes, activate a complete autophagy cascade, protect mitochondrial integrity, and alleviates hepatocyte injury in MASLD (127). Huazhi Rougan Granule can activate the SIRT1/AMPK pathway, enhance autophagic flux and lysosomal function, and reduce hepatocyte steatosis caused by FFAs (128).

#### 6.1.4 Nano-drug delivery systems

The nano-drug delivery system enhances the therapeutic effect by achieving targeted delivery of ALP modulators, thereby increasing the hepatic enrichment rate and reducing the off-target effect (129). The ROS-responsive nanoparticle system loaded with the autophagy activator Torin1 can target lysosomes, enhance lysosomal function through the TFEB pathway, and promote the effective degradation of lipids in MASLD (130). The sequential delivery system loaded with autophagy inhibitors can inhibit the formation of autolysosomes by increasing the pH value of autophagosomes and lysosomes, thereby inhibiting the proliferation and activation of HSC and alleviating liver fibrosis (129). As a new type of lysosomal regulator, attapulgite can be effectively internalized into the acidic lysosomal compartment, thereby restoring lysosomal acidification, activating autophagic flux and promoting substrate degradation, showing a protective effect in MASLD model (110). The nano drug delivery system is still in the preclinical stage; further breakthroughs are needed to address the challenges of large-scale production and clinical transformation.

#### 6.1.5 Targeting the gut-liver axis to modulate ALP

The gut-liver axis represents an emerging research field in MASLD therapy targeting ALP. HFD-fed mice and MASLD/MASH patients showed significant gut microbiota dysbiosis, especially the increased abundance of H2S-producing bacteria (such as Desulfovibrio). Studies have shown that hydrogen sulfide derived from the microbiota can induce autophagic cell death of c-Kit cDC1 cells in MASH patients and cause liver inflammation, which directly links intestinal flora imbalance with ALP dysfunction (87).

Probiotic and postbiotic strategies provide complementary intervention approaches. Extracellular vesicles derived from Lactobacillus salivarius SNK-6 can promote hepatocyte mitophagy, repair intestinal barrier and regulate gut microbiota composition through Beclin-1/PPAR pathway. These effects work together to improve systemic metabolism and reduce hepatic lipid accumulation (131). The convergence of the mechanism of action of probiotics on ALP provides a theoretical basis for incorporating gut microbiome regulation into ALP targeted therapy strategies. This view points out that ALP dysfunction is not only due to the defects of hepatocytes themselves, but also may be affected by distal factors from the gut, thus adding a new dimension to the theoretical framework of “multiple parallel hits”.

### 6.2 Non-pharmacological interventions

In addition to pharmacological agents, non-pharmacological interventions that regulate ALP function, such as dietary intervention, exercise, and lifestyle modification,serve as complementary strategies for MASLD management, with the advantages of high safety and convenient implementation (Table 2).

**Table 2:** Non-pharmacological intervention: targeting the autophagy-lysosomal system for MASLD.

Intervention Category	Specific Intervention	Developmental Stage / Investigational Models	Core Target & Regulated Pathway	Main Therapeutic Effects	References
Dietary Intervention	Mediterranean diet	Multiple human randomized controlled clinical trials (RCTs); In vivo: HFD-fed MAFLD mouse model	Activate AMPK/TFEB pathway; promote TFEB nuclear translocation and ALP-related gene transcription	Improve liver pathology in MASLD patients; activate ALP, enhance lipophagy and lysosomal function	(132–134)
	Intermittent fasting	In vitro: FFA-induced steatotic hepatocyte model; In vivo: HFD-fed MASH mouse / MASLD rat model	Restore autophagic flux; upregulate LAMP1, hepatic LAL activity and LAMP2 translocation to autolysosomes	Improve HFD-induced MASH; enhance triglyceride degradation, fatty acid oxidation and VLDL secretion	(135, 136)
Exercise Intervention	Long-term exercise/Chronic aerobic exercise	In vivo: HFD-fed MASH rat / MASLD mouse model	Downregulate FABP1 signaling; inhibit AKT-mTOR pathway; reduce ROS levels; inhibit cathepsin B release	Restore lysosomal acidification/proteolytic function; activate autophagy; reduce hepatic lipid accumulation, inflammation and mitochondria-dependent apoptosis; improve MASLD/MASH	(137, 138)
	Exercise-induced WDR41 lactylation	Human cohort correlative analysis; In vitro and in vivo: WDR41-depleted cells and mice	Lactylate WDR41 at K319→directly bind ATG13 and FIP200→drive ULK1 kinase complex assembly→initiate autophagy	Improve insulin sensitivity and lipid metabolism; negatively correlated with MASLD severity	(139)
	Resistance exercise	Human large-scale clinical cohorts and RCTs; In vitro and in vivo studies	Activate SIRT1; enhance lipophagy; regulate ALP	Improve hepatic lipid metabolism in MASLD	(140)
Lifestyle Modification	Weight control	Human large-scale clinical cohorts and RCTs	Elevate lysosomal acidification and LAL activity; relieve autophagic flux blockade	Reduce liver fat content and hepatic lipid accumulation; improve ALP function in MASLD patients	(77, 141)
	Smoking and alcohol cessation	Human large-scale clinical cohorts and RCTs; In vitro and in vivo studies	Reduce liver injury; prevent lysosomal dysfunction; maintain ALP homeostasis	Protect liver function and maintain normal ALP activity in MASLD	(142)

#### 6.2.1 Dietary intervention

Diet intervention is the basic strategy of MASLD management, and its core is to reduce lipid accumulation by adjusting dietary structure (132). The Mediterranean diet rich in unsaturated fatty acids in olive oil, fish and nuts can significantly improve the liver lesions of MASLD patients; this mechanism is closely related to the activation of ALP and the enhancement of lipophagy (133). This diet can activate the AMPK/TFEB pathway, promote TFEB transport to the nucleus, ALP-related gene transcription, lysosomal function and lipophagy (134). Intermittent fasting can enhance hepatic autophagy-lysosome function by restoring autophagic flux and upregulating LAMP1 expression, thereby improving MASH lesions caused by HFD (135). In addition, fasting can also increase hepatic LAL activity and promote the transfer of LAMP2 to autophagic lysosomes, thereby enhancing triglyceride degradation, fatty acid oxidation and very low-density lipoprotein (VLDL) secretion (136).

#### 6.2.2 Exercise intervention

Exercise intervention can improve the pathological state of MASLD by regulating energy metabolism and activating ALP. Long-term exercise can down-regulate the signal transduction of fatty acid binding protein 1 (FABP1), restore lysosomal acidification and proteolysis to enhance autophagic flux, thereby reducing hepatic lipid accumulation and improving MASLD (137). Chronic aerobic exercise can inhibit the AKT-mTOR pathway to activate autophagy, reduce the level of ROS to alleviate the increase of lysosomal membrane permeability, and inhibit the release of cathepsin B to the cytoplasm, thereby reducing inflammation and mitochondrial-dependent apoptosis, and ultimately improving NASH induced by HFD (138).

Recent breakthrough research reveals the molecular mechanism of exercise activating ALP: exercise can induce WDR41 protein to lactylate at lysine 319 site, and lactylated WDR41 promotes autophagy initiation by directly binding to ATG13 and FIP200, and drives ULK1 kinase complex assembly. This mechanism is essential for exerciseto improve insulin sensitivity, regulate lipid metabolism, and inhibit excessive lipid accumulation in the liver. Notably, the expression level of WDR41 was negatively correlated with the degree of lactonization in patients with multiple liver metabolic disorders, indicating that lactonized WDR41 is a key factor and potential biomarker in the pathogenesis of MASLD (139). Resistance exercise can also improve hepatic lipid metabolismby regulating ALP, which is mediated by SIRT1 activation and enhanced lipophagy (140).

#### 6.2.3 Lifestyle modification

Lifestyle modification is an important part of non-pharmacological interventions, including weight control, smoking cessation, alcohol restriction, and maintaining regular sleep patterns (141). Obesity is the main risk factor for MASLD. Weight loss can significantly improve hepatic lipid accumulation and ALP function. For MASLD patients, weight loss can reduce liver fat content, enhance lysosomal acidification and LAL activity, and alleviate autophagic flow block (77). Smoking cessation and alcohol restriction can reduce liver injury, prevent further damage to lysosomal function, and help maintain ALP homeostasis (142).

## 7 Conclusion and future perspectives

The ALP dysfunction in MASLD is not a single defect, but a dynamic, stage-dependent and cell type-specific process. There are four interrelated molecular levels: lysosomal acidification failure (beyond pH elevation, covering V-ATPase subunit-specific defects such as ATP6V1B2 and ATP6V0A1, and LAMTOR1-mediated mTORC1 dysregulation), autophagosome-lysosomal fusion blockade (involving Rab7, syntaxin 17, and SNARE complex abnormalities), transcriptional/epigenetic dysregulation (mTORC1 overactivation-mediated TFEB inhibition and NSD2-catalyzed H4K20me3 epigenetic silencing), and selective autophagy defects (lipophagy, mitophagy, and endoplasmic reticulum autophagy). These levels together constitute a self-reinforcing pathogenic network, in which lysosomal dysfunction is the core bottleneck, which weakens the degradation ability and hinders the fusion efficiency, and finally perpetuates the blocking of autophagic flow. The emerging interaction between ALP dysfunction and ferroptosis further consolidates these pathogenic processes, as impaired autophagy clearance allows lipid peroxidation substrates to accumulate, thereby driving ferroptosis cell death, while TFEB-mediated lysosomal enhancement in Kupffer cells confers resistance to ferroptosis through NADPH generation. This integrated framework positions ALP as a unified pathogenic hub, amplifying multiple parallel hits throughout MASLD—lipid overload, oxidative stress, endoplasmic reticulum stress, and mitochondrial damage.

The functional trajectory of ALP in the progression of MASLD reveals a key shift from compensatory activation in simple steatosis to clear blockage in MASH and fibrosis, accompanied by cell type-specific duality, in which hepatocytes exhibit impaired protective autophagy, while HSCs exhibit enhanced pro-fibrotic autophagy. This stage-dependent and cell type-specific feature has far-reaching therapeutic implications: ALP activation strategy is most suitable for MASH patients with established autophagic flux blockade, and indiscriminate activation in early steatosis may be unnecessary. In addition, the opposite autophagy activity between hepatocytes and HSCs requires selective intervention of cell types—such as intervention using the RAB18 phase separation mechanism—rather than global pathway regulation. The identification of epigenetic regulators such as NSD2 and gut-liver axis mediators (microbiota-derived H2S, probiotic extracellular vesicles) adds additional therapeutic complexity and opportunities. These insights collectively require accurate patient stratification based on disease stage, hepatocyte type participation, and ALP functional status, and promote the field to a personalized treatment strategy for specific molecular lesions of ALP failure in each patient.

Although substantial progress has been made in research, several key challenges still need to be addressed to translate mechanism findings into clinical practice. The long-term safety of ALP activation in the context of HCC risk remains a concern, as once malignant tumors are established, autophagy can support tumor cell survival—an “autophagy paradox” that requires an appropriate therapeutic window at the stage. It is urgent to develop non-invasive biomarkers that can accurately reflect liver lysosomal acidification, autophagic flux and selective autophagy activity to achieve patient stratification and treatment monitoring. The increasing understanding of ALP dysfunction as a dynamic, context-dependent and treatment-accessible process has laid a solid foundation for the development of stage-specific, cell type-selective and mechanism-guided treatment strategies that are expected to improve the clinical outcomes of millions of patients affected by MASLD and its complications.
